# Correlation of Eye Diseases with Odontogenic Foci of Infection: A Case Report Using Infrared Thermography as a Diagnostic Adjunct

**DOI:** 10.3390/healthcare13243283

**Published:** 2025-12-15

**Authors:** Daria Wziątek-Kuczmik, Aleksandra Mrowiec, Anna Lorenc, Maciej Kamiński, Iwona Niedzielska, Ewa Mrukwa-Kominek, Armand Cholewka

**Affiliations:** 1 Department of Cranio-Maxillofacial Surgery, Medical University of Silesia, 40-027 Katowice, Poland; 2 Faculty of Science and Technology, University of Silesia, 40-007 Katowice, Poland; 3 Department of Ophthalmology, Faculty of Medical Science, Medical University of Silesia, 40-514 Katowice, Poland

**Keywords:** odontogenic foci, maxillary sinusitis, intraocular pressure, uveitis, glaucoma, infrared thermography

## Abstract

**Introduction**: Odontogenic infections may influence distant structures, including the eye. Their extension into the paranasal sinuses and orbital region can contribute to inflammatory and glaucomatous conditions. **Case Report**: A 46-year-old man was examined for a possible odontogenic source of chronic eye disease. The patient had an 18-year history of progressive vision loss in his left eye associated with Posner–Schlossmann syndrome, chronic uveitis, and episodic elevation of intraocular pressure (IOP). Imaging studies revealed the presence of a foreign body in the alveolar recess of the left maxillary sinus, as detected on panoramic radiography, cone-beam computed tomography (CBCT), and infrared thermography (IRT). Preliminary IRT examination showed marked thermal asymmetry (ΔT = 1.1 °C) between the left and right sides of the maxilla. Worsening of ocular symptoms and increased IOP despite steroid treatment prompted surgical treatment. The foreign body, identified as a root canal filling, was removed, and the chronically inflamed sinus mucosa was excised. During a follow-up visit two weeks later, the IRT examination showed a reduction in temperature difference (ΔT = 0.2 °C) and routine postoperative healing. After two months, no thermal asymmetry was found (ΔT = 0 °C), and an ophthalmological examination showed no active inflammation. Six months after the procedure, the patient remained asymptomatic, and the IRT examination revealed only minimal residual variability within the measurement tolerance (ΔT = 0.1 °C), consistent with the resolution of the sinus inflammation. **Conclusions**: This case highlights the value of interdisciplinary diagnostics in identifying odontogenic contributors to chronic ocular disease. Infrared thermography proved to be a helpful non-invasive adjunct for detecting and monitoring subclinical maxillary sinus inflammation.

## 1. Introduction

Odontogenic foci of infection can affect a person’s general health. Among diseases pathogenetically associated with infections in the oral cavity are diseases of the organ of vision, such as iritis, keratitis, and optic neuritis [1,2,3,4]. Dental pathologies leading primarily to odontogenic maxillary sinusitis spreading to the orbital space can result in serious complications such as vision loss and cavernous sinus thrombosis; they can be life-threatening [1,4,5]. The publication describes an unusual case in which chronic uveitis and secondary glaucoma occurred because of inflammation of the paranasal sinuses due to odontogenic complications. This report presents a rare case of ocular inflammation associated with an odontogenic focus in the maxillary sinus, highlighting the role of infrared thermography as a complementary diagnostic tool in identifying subclinical infections.

## 2. Case Presentation

### 2.1. Interview

An ophthalmologist referred a 46-year-old Caucasian man to the Oral and Maxillofacial Surgery Clinic to rule out odontogenic foci of infection. He reported no oral symptoms. He reported progressive deterioration of vision in his left eye. He associated his ocular symptoms with endodontic treatment of his upper left first molar 18 years earlier. For unknown reasons, the tooth was extracted several years after the treatment was completed. Approximately 10 years later, a surgical procedure was performed at the Otolaryngology Department to remove a foreign body from the left maxillary sinus, diagnosed based on computed tomography (CT) of the paranasal sinuses. The procedure was not radical, and the patient was not informed of this (no medical records were available). During repeated hospitalizations at the Ophthalmology Clinic and extensive testing, including serological testing for autoimmune and infectious diseases (ANA, CRP, VDRL, HIV, and parasite screening), no systemic abnormalities were found. Posner–Schlossmann glaucoma and chronic uveitis were diagnosed in the left eye. Persistently elevated intraocular pressure (IOP) led to advanced optic neuropathy. To control the IOP, a trabeculectomy with paracentral iridectomy was performed. Despite multiple courses of anti-inflammatory, antiviral, and antihypertensive therapy, as well as transscleral diode laser cyclophotocoagulation (TSCPC), the IOP remained elevated.

The lack of treatment effects and the progressive deterioration of vision in the left eye significantly impacted the patient’s mental health. This prompted the patient to see a psychiatrist, who diagnosed depression and recommended antidepressant treatment.

### 2.2. Dental Diagnostic Examination

The dental examination revealed good oral hygiene and the absence of one upper left molar in the maxillary arch. The oral mucosa covering the alveolar process was intact and without signs of inflammation.

A panoramic X-ray revealed an unshadowed alveolar recess in the left maxillary sinus with a central, irregular shadow in the previously extracted tooth 26. To thoroughly assess the left maxillary alveolar recess, a CBCT scan was performed, which located a foreign body with good contrast. This image suggested the presence of endodontic material used to fill the root canal.

**Figure 1:**
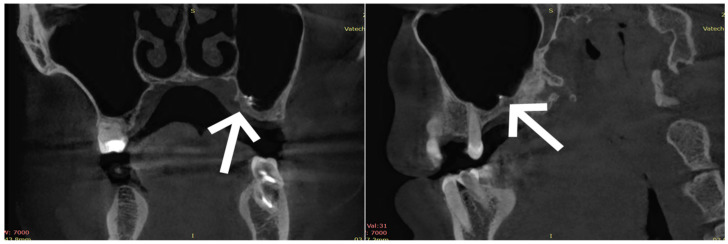
A CBCT scan revealed a radiopaque foreign body in the left maxillary sinus (arrow).

Cone beam computed tomography (CBCT) (Figure 1) Reconstructions were performed with an isotropic voxel size of 0.2 × 0.2 × 0.2 mm, with an in-plane field of view of approximately 12 × 12 cm (600 × 600 pixels, pixel spacing 0.20 mm) confirmed the presence of a foreign body, accompanied by a slight inflammatory reaction, in the alveolar recess of the left maxillary sinus. The patient was scheduled for a thermographic examination, as per established algorithms, to complete the diagnostic process.

The diagnostic workflow was supplemented by thermographic imaging in accordance with the established protocol.

Four thermographic measurements were obtained using a FLIR T540 thermal imaging camera, following the pathology identified on CBCT examination. The imaging was performed in a controlled environment at 23 ± 1 °C and 50% relative humidity, with an emissivity setting of 0.98. The camera is equipped with an uncooled microbolometer detector with a 17-μm pixel pitch, a thermal sensitivity (NETD) of <30 mK at 30 °C, and a native thermal resolution of 464 × 348 pixels. The manufacturer-reported accuracy of ±2 °C or ±2% of the reading was considered when interpreting ΔT values.

The camera was positioned at a distance of 0.4 ± 0.05 m, within the minimum focus range for the 42° lens (0.15 m) and aligned perpendicular to the patient’s facial midline to minimize geometric distortion. Anatomical landmarks, including the zygomatic eminences, nasolabial folds, infraorbital rim, and midline, were used to ensure consistent head orientation [6]. The patient sat with the head stabilized in a reproducible position and the mouth open. Before imaging, the patient rinsed the oral cavity with room-temperature water for approximately one minute, followed by a 20-min acclimatization period to reduce physiologic and environmental variability such as changes in skin perfusion, residual activity effects, circadian fluctuations, or transient mucosal Warming [6,7]. Environmental temperature and humidity were continuously monitored to control for thermal drift. Each acquisition was performed after a 30-s stabilization period with the mouth open.

The region of interest (ROI) corresponded to the cutaneous projection of the alveolar recess of the maxillary sinus containing the foreign body, as well as the symmetrical area on the contralateral side. The ROIs were defined by a maxillofacial surgeon based on radiographic landmarks and clinical evaluation. The same operator delineated all ROIs to minimize inter-rater variability; intra-rater consistency was verified by repeating ROI placement three times on baseline thermograms, yielding differences <0.1 °C, well below the camera’s thermal sensitivity. Mean temperature values within each ROI were used for analysis.

Differences in bilateral temperature (ΔT) were interpreted with reference to device accuracy, NETD, and expected physiologic symmetry under controlled conditions. The pre-operative ΔT of 1.1 °C substantially exceeded the typical bilateral variability observed in healthy individuals under standardized thermographic protocols [6]. Subsequent reductions (0.2 °C at two weeks and 0.0–0.1 °C at later follow-ups) were interpreted cautiously, acknowledging that values within ±0.1 °C approach the camera’s noise floor; for this reason, ΔT values after the second week were considered thermographically negligible.

Although the mucosa of the maxillary sinus is not externally visible, thermal differences may still be detectable through cutaneous projection due to alterations in local blood flow and perfusion of overlying soft tissues. This principle is widely used in thermographic evaluation of deeper inflammatory processes; however, attenuation by soft tissues and individual perfusion variability represent inherent limitations. Therefore, strict environmental control, bilateral comparison, and repeated measurements were essential for reliable interpretation [7]. The relatively thin skin in the infraorbital region (typically 1–2 mm) and the proximity of the alveolar recess to the facial surface support the feasibility of detecting small thermal gradients. However, absolute temperature values should be interpreted with caution.

### 2.3. Treatment

The patient was qualified for endoscopic surgery of the left maxillary sinus. The surgery involved removing the foreign body, which was identified as a root canal sealant used in endodontic treatment, and removing the macroscopically inflamed mucosa from the left maxillary sinus cavity.

The foreign body was only subjected to macroscopic examination. Intraoperatively, the iatrogenic material was grayish in color, with a slightly porous surface, and was surrounded by thickened mucosa. The surrounding mucosa was subjected to histopathological evaluation, and the results revealed: polypoid fragments of the mucosa, connective tissue, and a small fragment of squamous epithelium.

### 2.4. Outcome and Follow-Up

**Figure 2:**
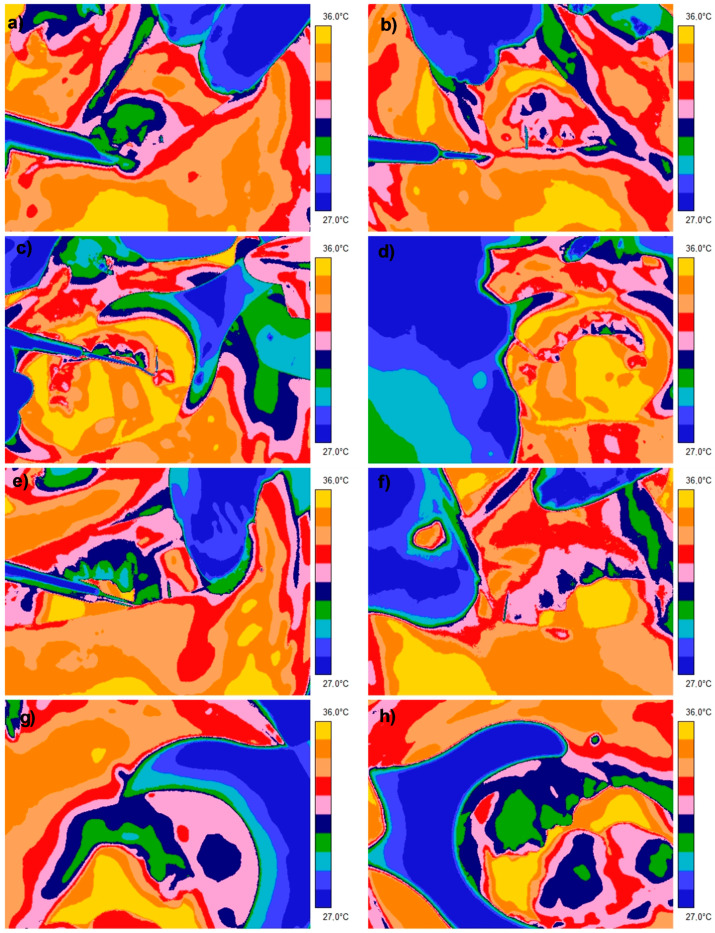
Representative thermal images of all four examinations: (**a**) Baseline thermogram with foreign body in the left maxillary sinus. (**b**) Contralateral control at baseline. (**c**) Thermogram 2 weeks postoperatively. (**d**) Contralateral control at 2 weeks. (**e**) Thermogram 2 months postoperatively. (**f**) Contralateral control at 2 months. (**g**) Thermogram 6 months postoperatively. (**h**) Contralateral control at 6 months.

The initial thermographic examination (I) of the alveolar recess of the maxillary sinus, corresponding to the presence of the foreign body, showed a significant difference compared to the symmetrical area on the right side of the maxilla, namely 1.1 degrees (Table 1; Figure 2a,b). At the next visit, the patient provided a medical certificate indicating an exacerbation of the underlying disease and increased intraocular pressure despite steroid therapy. The correlation between the clinical image and the thermographic one prompted the maxillofacial surgeons to qualify the patient for surgery on the left maxillary sinus. The surgery involved removing the foreign body, which was identified as a root canal sealant used in endodontic treatment, and removing the macroscopically inflamed mucosa from the left maxillary sinus cavity.

At the postoperative follow-up, 2 weeks after the surgery, proper healing was observed. A control thermographic examination (II) of the examined area compared to the reference showed a difference of 0.2 °C (Table 1; Figure 2c,d). At the second postoperative check-up, the patient provided a certificate from an ophthalmologist indicating that there were no signs of active inflammation in the left eye. The histopathological diagnosis of the material taken from the maxillary sinus indicated a chronic, polypoid inflammatory process.

**Figure 3:**
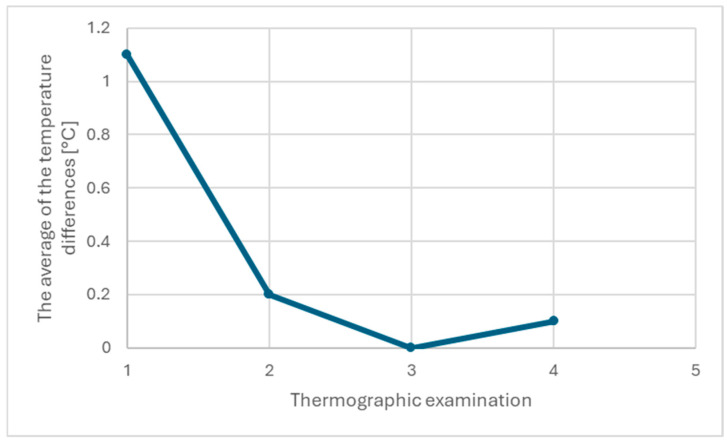
Average temperature differences in the maxillary sinus area before treatment and at 2 weeks, 2 months, and 6 months after the procedure.

To confirm the elimination of the inflammatory factor in the maxillary sinus cavity, a third thermographic examination (III) of the examined area compared to the reference was performed, where no temperature difference between the areas was observed (Table 1; Figure 2e,f). Another check-up and fourth thermographic measurement, conducted six months after the surgery, showed a difference of 0.1 °C (within error tolerance), no local complaints, complete nasal patency, and improved psychological well-being (Table 1; Figure 2g,h and Figure 3).

### 2.5. Ophthalmological Results and Long-Term Follow-Up

The intraocular pressure (IOP) was measured using an iCare tonometer, which quickly and painlessly obtained the result by measuring the force of the probe tip’s reflection from the cornea’s surface.

Additionally, measurements were taken using the Goldman tonometer application method. This is a contact method that requires corneal anesthesia. It uses the Imbert and Fick rule, in which the pressure inside the sphere is equal to the force needed to flatten it, divided by the size of the flattened surface. During the measurement, a particular prism is used to model and flatten the cornea’s surface, and a tonometer is also used. Before the examination, the cornea’s surface is anesthetized with Alcaine and stained with fluorescein. The light in the slit lamp is set to a blue color. In this way, an image of two semicircles is obtained, and then the tonometer scale is set so that the semicircles touch each other with their inner edges, allowing the pressure value to be read on the scale.

The patient’s local condition was not deteriorating during the eighteen-month follow-up period. In the follow-up one year after the procedure, the intraocular pressure was normalized only with topical medications, and oral sulfonamide was discontinued. The clinical condition improved significantly.

The patient remained on only local steroid therapy, anti-inflammatory drugs, and medications lowering intraocular pressure.

The eyeball showed no inflammation, and the intraocular pressure was 16 mmHg.

**Figure 4:**
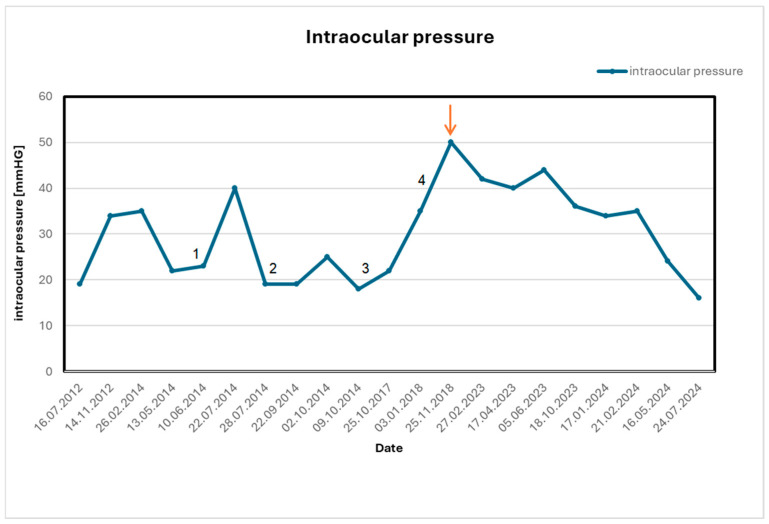
Graph showing intraocular pressure measurements of the left eye (oculus sinister—OS) throughout the entire ophthalmic therapy. The orange arrow indicates the date of maxillary sinus surgery. The measurement was performed primarily using the Goldman method, with the Icare method also used in individual cases. Nr 1—TSCPC OS and anti-inflammatory treatment, nr 2—trabeculectomy with iridectomy OS and anti-inflammatory treatment, nr 3—cataract surgery OS and anti-inflammatory treatment, nr 4—TSCPC OS and anti-inflammatory treatment.

Figure 4 shows an apparent decrease in intraocular pressure after the surgical procedure, indicating the treatment’s effectiveness.

The normal range of intraocular pressure is 10–21 mmHg, which is crucial for maintaining the eye’s anatomical shape and the proper functioning of the visual organ.

## 3. Discussion

According to a report published by the CareQuest Institute for Oral Health, visual impairment and systemic causes of ocular problems, including diabetes and heart disease, are associated with poor oral health [8]. Therefore, a dental examination with a complete radiological diagnosis is essential to interdisciplinary medical care. This case raises an important issue: the correlation of ocular disorders with the emphasis on dentistry.

Given the anatomical relationships of the orbital tissues, infections of the maxillary sinus can spread rapidly. They cause severe orbital inflammation and carry the risk of vision loss and even life-threatening consequences. This tendency can be attributed to the adjacent paranasal sinuses and orbital contents, separated by a thin bony wall called the papyracea [9].

Asymptomatic foreign bodies lodged in the lumen of the maxillary sinus can be a source of bacterial, toxic, allergic, or neural obstruction. Through bacterial dissemination, bacterial toxin secretion, or allergic reaction, they can damage the organ of vision [10]. Structural and functional changes of the optic nerve correlate with the severity of chronic sinusitis. There are also sporadic reports of optic neuropathy caused by mechanical compression of the optic nerve, impaired circulation of nerve vessels caused by mechanical compression, and optic neuritis caused by inflammatory conditions such as polyps, invasive sinonasal aspergillosis, acute bacterial sphenoid sinusitis, mucous eosinophilic sinusitis, allergic fungal sinusitis, and sinusitis adjacent to the optic nerve [9,10,11,12].

Available population-based cohort studies in Taiwan demonstrate that optic nerve (ON) damage in patients with chronic rhinosinusitis (CRS) is not uncommon. There may be a causal relationship between the development of ON and the presence of CRS, which has rarely been previously elucidated. Studies have confirmed that inflammatory or infectious lesions of CRS can infiltrate and affect nearby tissues, contributing to orbital apex syndrome and damage to the optic nerve. The authors demonstrated that the presence of CRS is a significant risk factor for the development of ON. Furthermore, the risk of developing ON is positively increased, particularly in CRS patients who have previously undergone FESS (endoscopic sinus surgery) [12]. Although the sphenoid sinus is considered the most common source of inflammation due to its proximity to the optic nerve, ocular complications related to maxillary sinus disease are not uncommon. Unilateral maxillary sinusitis carries a risk of serious complications such as optic neuritis, meningitis, or cavernous sinus thrombosis [13].

If no results are achieved after many months of high-dose steroid therapy, rhinogenic optic neuropathy should be considered, and multidisciplinary diagnostics should be implemented.

Therefore, orbital inflammation should be evaluated through a multidisciplinary examination involving ophthalmology, dentistry, and laryngology. In our case, the maxillofacial surgeon combined the disciplines of dentistry and laryngology. A non-infectious factor or an infection could have caused the uveitis observed in the patient. In inflammation of the anterior segment of the uvea, characteristic deposits on the endothelium and inflammatory cells in the anterior chamber suspended in the aqueous fluid were observed during slit-lamp examination. Uveitis can manifest as inflammation of the anterior, intermediate, or posterior segment of the eyeball. The incidence of uveitis is estimated at 38 cases per 100,000 people [14,15]. It is a rare condition, and diagnosing it is not easy. Most uveitis occurs in conjunction with non-infectious systemic diseases, which were excluded in the patient.

The literature on uveitis as a complication of sinusitis is very scarce. Saccà SC et al. describe an unusual case of an 11-year-old child in whom bilateral sinusitis was accompanied by bilateral uveitis complicated by bilateral papillitis of the optic nerve, which entirely resolved after appropriate treatment of sinusitis. Initial conservative treatment, steroids, and antibiotic therapy brought immediate but short-term effects. After 2 months, inflammation recurred. Only laryngological surgery brought a cure [16]

In the presented case, microextrusion of the endodontic material or irrigant (sodium hypochlorite, formaldehyde, eugenol) beyond the root apex, and toxin penetration into the periapical tissues, maxillary sinus, and, rarely, the orbit, cannot be ruled out. This resulted in slow chemical inflammation and nerve fiber damage [17,18]

Based on our own and the cited descriptions, we assume the following mechanism: sinusitis causes severe inflammation, resulting in the massive release of proinflammatory cytokines, which reach the orbit and involve the pre-lamellar part of the optic nerve. Common complications of ocular inflammation, such as retinal edema, may be due to the action of cytokines, which can induce edema in the optic nerve. Studies on the role of cytokines in uveitis support our hypothesis [12,19].

In the present case, normalization of ocular inflammation and intraocular pressure occurred after surgical removal of the sinus foreign body. This *temporal association* raises the possibility that maxillary sinus inflammation contributed to the ocular manifestations; however, a causal relationship cannot be established in a single case, particularly in the context of multimodal ophthalmic therapy, fluctuating disease activity in Posner–Schlossman syndrome, and the known effects of corticosteroids on IOP. Therefore, the observed improvement should be interpreted as a correlation that is *hypothesis-generating* rather than confirmatory. To clarify the inference hierarchy in this case, we outline the main causal and non-causal pathways that could explain the observed postoperative improvement. In a conceptual diagram of the anatomical relationships, the suspected odontogenic focus could influence ocular inflammation either directly (via inflammatory mediators extending from the maxillary sinus to the orbit) or indirectly (via systemic cytokine release). However, several alternative pathways may equally or better explain the outcome. (1) Natural variability/regression to the mean: Posner–Schlossman syndrome is characterized by spontaneous, episodic fluctuations in intraocular pressure and uveitis activity, independent of external intervention. (2) Concurrent therapy: The patient continued topical steroids, anti-inflammatory agents, and pressure-lowering medications; improvements may reflect pharmacological effects rather than sinus surgery. (3) Steroid-related IOP dynamics: Systemic or topical steroids can both raise and normalize IOP depending on dose and timing, introducing additional complexity. (4) Post-surgical systemic changes: Reduced stress, improved sinonasal airflow, or nonspecific postoperative immune modulation may have contributed to symptom improvement. Because these alternative pathways can lead to the same observed outcome, the available evidence supports only a temporal association between surgical treatment and ocular improvement, not a definitive causal link. Another rare ophthalmological disorder diagnosed in the patient is Posner-Shlossman syndrome, characterized by recurrent episodes of increased intraocular pressure accompanied by symptoms of uveitis. It is usually a unilateral disorder in adults and young adults aged 20–50 years [19]. The cause of this syndrome is unknown, although some researchers associate it with viral infections, including CMV, VZV, and HSV. In viral infections, it is possible to induce an inflammatory process in the trabecular meshwork, which may cause swelling and insufficiency of the eye angle [20,21,22]. It is also likely related to vascular abnormalities, including abnormal endothelium-dependent vasodilation. Episodes of increased intraocular pressure may also be related to inflammatory factors and active inflammatory processes [20,23]. The described case raises yet another systemic aspect, namely, mental health and chronic inflammatory processes. Depression and anxiety are often undiagnosed in patients with chronic diseases such as sinusitis, probably because physicians are usually unaware of the significance of depression and anxiety in the treatment of primary diseases. In addition, chronic use of systemic corticosteroids in patients with sinusitis and ophthalmological conditions may lead to the development of depression [24,25,26,27].

Infrared thermography has been utilized to detect asymptomatic foci of infection within the maxillary sinus lumen, providing a valuable complement to conventional diagnostic imaging methods, including X-rays and computed tomography. This test is non-invasive and non-contact, involving the acquisition and analysis of thermal maps using thermal imaging cameras. Thermography non-invasively records changes in body temperature, which may correlate with blood supply and tissue metabolism [28,29,30]. Thermography is useful for initially diagnosing silent dental foci in patients at high risk of systemic infection [31]. The use of thermographic imaging in the diagnosis of maxillary sinus inflammation deserves special attention [31,32,33].

According to recent review reports, dental procedures can cause ocular complications, particularly neuro-ophthalmic complications, including cranial nerve palsies and blindness. The process leading to ophthalmological complications may be multifactorial, and the impact of concomitant infections of the paranasal sinuses remains unclear [18].

This case supports the hypothesis that odontogenic inflammation, even when clinically silent, can contribute to ocular pathology by releasing inflammatory mediators. Early identification and surgical management of such foci may prevent irreversible ocular complications.

## 4. Conclusions

This case highlights the importance of identifying the sources of odontogenic and sinus infections in patients with atypical or treatment-resistant ocular conditions. Interdisciplinary collaboration between ophthalmologists, dentists, and maxillofacial surgeons is essential. Infrared thermography can provide a noninvasive complement to the diagnosis of subclinical inflammatory lesions.

## Figures and Tables

**Table 1:** Average temperature values measured over a 30-s window after mouth opening, obtained before treatment (Measurement I) and at 2 weeks (Measurement II), 2 months (Measurement III), and 6 months (Measurement IV).

Measurement	Maxillary Sinus	Average Temperature [°C] at 30 s
**The first measurement**	Left maxillary sinus	36.1
	Right maxillary sinus	35
	Difference average temperaturę	1.1
**The second measurement**	Left maxillary sinus	35.2
	Right maxillary sinus	35
	Difference average temperature	0.2
**The third measurement**	Left maxillary sinus	34.3
	Right maxillary sinus	34.3
	Difference average temperature	0
**The fourth measurement**	Left maxillary sinus	32.5
	Right maxillary sinus	32.4
	Difference average temperature	0.1

## Data Availability

The raw data supporting the conclusions of this article will be made available by the authors on request.
